# The Medical College Question

**Published:** 1887-03

**Authors:** 


### THE MEDICAL COLLEGE QUESTION.

Texas is filling up at the rate of 200,000 immigrants annually. Ere many years, all the vacant lands and waste places will be filled up, and what is now " desert, will blossom as the rose." The enterprise and capital of live men in Europe and America are directed to the development of the rich resources of this wonder land—resources scarcely touched as yet, and in a period of ten years—about the time, it is estimated by the Regents, that will be required to make the present resources of the University available for a medical department, such development and growth will have taken place as to change entirely the present aspect of things. (In this connection we may be pardoned for mentioning a fact, significant of this development, and as illustrating our idea. The cost of fuel has been a serious drawback to many Texas cities—notably, Fort Worth and Austin. Coal has sold here till the present time at $8.50

per ton. The opening of adjacent coal fields recently, has reduced the price to $5.50.)

The growth of our principal cities has been *pari-passu* with this influx into the State; and tho' Galveston had fairly the start, and now boasts superior clinical advantages, as *the* argument in favor of the separate establishment of a Medical Department, it is highly probable,—nay—almost absolutely certain, that by the time, in the very nature of things, that a medical college could be put into successful operation, she will have rivals in that field. We make no claim for Austin, other than that we are in favor of consolidating all the departments under one management—all things being equal —i. e., should Austin, by the time we speak of, possess anything like the clinical advantages offered by other cities.

And, with regard to the munificent offer of the city of Galveston and of the late Mr. Sealy, we will be much mistaken if, when the time arrives for action, some such offer is not made by Austin and some of her wealthy citizens, and by other cities also. Nearly any city would give a bonus for the college. We recall the circumstance a few years ago when this subject was being discussed with reference to Fort Worth. The mayor gave the assurance, and it was no empty one, that immediately on the completion of a college building in that city, a $50,000 hospital would be built adjoining it; while several citizens offered building sites; and a subscription paper started, soon made it evident that funds could be raised amongst the business men sufficient to build the college.

With regard to the question of unconstitutionality of the act locating the medical department at Galveston we will speak anon.

